# Epicardial and endocardial surgical ablation of atrial fibrillation: outcomes from CASE-AF Registry

**DOI:** 10.1093/icvts/ivae123

**Published:** 2024-06-27

**Authors:** Ivana Mitrovic, Edgar Eszlari, Adi Cvorak, Andreas Liebold, Ardawan Rastan, Herko Grubitzsch, Michael Knaut, Theodor Fischlein, Taoufik Ouarrak, Jochen Senges, Thorsten Hanke, Nicolas Doll, Walter Eichinger

**Affiliations:** Department of Cardiac Surgery, Munich Clinic Bogenhausen, Munich, Germany; Department of Cardiac Surgery, Munich Clinic Bogenhausen, Munich, Germany; Department of Cardiac Surgery, Munich Clinic Bogenhausen, Munich, Germany; Department of Cardiothoracic and Vascular Surgery, Ulm University Hospital, Ulm, Germany; Department of Cardiothoracic and Vascular Surgery, Philipps-University Hospital, Marburg, Germany; Department of Cardiothoracic and Vascular Surgery, Charite University Hospital, Berlin, Germany; Department of Cardiac Surgery, University Hospital Carl Gustav Carus, Dresden, Germany; Department of Cardiac Surgery, Nuremberg Clinic, Paracelsus Medical University, Nuremberg, Germany; Institute for Heart Attack Research, Ludwigshafen, Germany; Institute for Heart Attack Research, Ludwigshafen, Germany; Department of Cardiac Surgery, Asklepios Clinic Harburg, Hamburg, Germany; Department of Cardiac Surgery, Schuechtermann-Clinic, Bad Rothenfelde, Germany; Department of Cardiac Surgery, Munich Clinic Bogenhausen, Munich, Germany

**Keywords:** Atrial fibrillation, Radiofrequency, Cryoablation, Concomitant surgical ablation

## Abstract

**OBJECTIVES:**

The German CArdioSurgEry Atrial Fibrillation Registry is a prospective, multicentric registry analysing outcomes of patients undergoing surgical ablation for atrial fibrillation as concomitant or stand-alone procedures. This data sub-analysis of the German CArdioSurgEry Atrial Fibrillation Registry aims to describe the in-hospital and 1-year outcomes after concomitant surgical ablation, based on 2 different ablation approaches, epicardial and endocardial surgical ablation.

**METHODS:**

Between January 2017 and April 2020, 17 German cardiosurgical units enrolled 763 consecutive patients after concomitant surgical ablation. In the epicardial group, 413 patients (54.1%), 95.6% underwent radiofrequency ablation. In the endocardial group, 350 patients (45.9%), 97.7% underwent cryoablation. 61.5% of patients in the epicardial group and 49.4% of patients in the endocardial group presenting with paroxysmal atrial fibrillation. Pre-, intra- and post-operative data were gathered.

**RESULTS:**

Upon discharge, 32.3% (*n* = 109) of patients after epicardial surgical ablation and 24.0% (*n* = 72) of patients after endocardial surgical ablation showed recurrence of atrial fibrillation. The in-hospital mortality rate was low, 2.2% (*n* = 9) in the epicardial and 2.9% (*n* = 10) in the endocardial group. The overall 1-year procedural success rate was 58.4% in the epicardial and 62.2% in the endocardial group, with significant symptom improvement in both groups. The 1-year mortality rate was 7.7% (*n* = 30) in epicardial and 5.0% (*n* = 17) in the endocardial group.

**CONCLUSIONS:**

Concomitant surgical ablation is safe and effective with significant improvement in patient symptoms and freedom from atrial fibrillation. Adequate cardiac rhythm monitoring should be prioritized for higher quality data acquisition.

**Figure:**
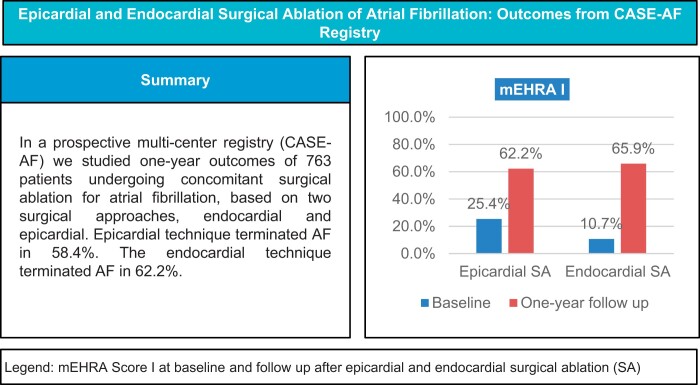

## INTRODUCTION

Atrial fibrillation (AF) is the most common abnormal rhythm of the heart, occurring in 5–41% of the patients who undergo cardiac surgery, depending on the underlying disease [1]. As an arrhythmia associated with an elevated risk of stroke, heart failure and diminished quality of life, it continues to drive the essential quest for effective treatment strategies. Surgical ablation techniques have evolved significantly since their inception, nearly 40 years ago. Novel technologies for AF therapy, which have simplified the Cox-maze III procedure create transmural atrial lesions using radiofrequency and cryo-energy and are proof of the advancements in ablative surgery. Endocardial and epicardial surgical ablation are 2 different approaches to treating AF. Each approach has its advantages and considerations, and the choice between them depends on the surgeon's expertise, the patient's specific condition and the overall surgical plan. The left atrial appendage (LAA) closure should be incorporated into concomitant surgical ablation today. According to current guidelines, surgical ablation is recommended for only class IIa indication, whereas LAA occlusion is recommended for class IIb indication for stroke prevention in patients with AF undergoing cardiac surgery [2]. Monitoring and re-evaluating the current standard of practice is paramount for patients who underwent surgical ablation during concomitant cardiovascular procedures. Due to the lack of data and information, the survey of real-world scenarios provides important information for decision-making in surgical ablation. The German CArdioSurgEry Atrial Fibrillation Registry (CASE-AF Registry), governed by the Institute for Heart Attack Research is an ongoing nationwide (Germany), prospective, observational, multicentre study with a focus on surgical ablation. For this study, we conducted a subgroup analysis within the CASE-AF Registry. Our primary aim was to describe in-hospital and 1-year outcomes by patients who underwent concomitant surgical ablation, based on the surgical approach employed, whether it is epicardial or endocardial surgical ablation.

## METHODS AND PATIENTS

### Ethical considerations

This study was approved by the Ethics Committee on 6 May 2016 [Landesärztekammer Rheinland-Pfalz, Germany, ID: 837.536.15 (10304)]. The written patient informed consent was obtained from each patient before surgical ablation.

### The German CArdioSurgEry Atrial Fibrillation registry

The CASE-AF Registry, with ClinicalTrials.gov identifier NCT03091452, registered on March 27, 2017, stands as the largest national registry devoted to collecting and describing the in-hospital and 1-year outcomes of patients following surgical ablation. Between January 2017 and April 2020, 17 cardiothoracic centres across Germany enrolled 1115 patients who underwent either stand-alone or concomitant surgical ablation. To participate, patients were required to be over 18 years old, provide signed consent and have a planned surgical ablation. In-hospital and 1-year outcomes of the CASE-AF Registry have already been published [3, 4]. Additionally, the 1-year outcomes of the CASE-AF Registry, particularly regarding surgical ablation for long-standing atrial fibrillation, have been published [5]. For our analysis, we excluded 2 specific groups of patients: those who underwent stand-alone and those who had both epicardial and endocardial surgical ablation simultaneously done. We divided the 763 remaining patients into 2 groups, the epicardial (*n* = 413, 54.1%) and the endocardial group (*n* = 350, 45.9%), based on the surgical approach.

### Definitions

The classification of AF into paroxysmal, persistent, or long-standing persistent was based on the 2016 ESC/EACTS Guidelines for the management of AF [6]. The modified European Heart Rhythm Association symptom classification for AF (mEHRA) was utilized at baseline and the 12-month follow-up to evaluate the severity of the patient's symptoms [7]. Early recurrence of AF was defined as any AF recurrence lasting longer than 30 seconds during the blanking period (3 months after ablation), while late recurrence of AF was characterized by any AF recurrence longer than 30 seconds after the blanking period and was defined as a procedural failure. Procedural success was defined as freedom from AF after the blanking period, as determined by 12-lead electrocardiogram (ECG) findings including Holter ECG and/or implanted devices, without the need for redo-ablative procedures, no further electric synchronized cardioversion, no rehospitalization due to AF, regardless of the use of antiarrhythmic drugs.

### Data analysis

Central data management and analysis were overseen by The Institute for Heart Attack Research in Ludwigshafen/Rhein, Germany. Utilizing an electronic case report form, they facilitated data upload and collection of preoperative, procedural and postoperative data as well as 1-year follow-up outcomes. Key data collected at the 1-year follow-up included arrhythmia documentation, rehospitalization, mEHRA classification, cardioversion procedures, re-do ablation, pacemaker implantation rates, postoperative complications, anticoagulant usage, antiarrhythmic drug administration and mortality. The Institute for Heart Attack Research conducted rigorous central data monitoring, addressing initial queries in the electronic case report form to ensure data accuracy.

Categorical variables were represented as counts and percentages and compared using the Chi-square test. The McNemar test assessed significant changes in symptom improvement within both groups. Continuous variables were compared using the Mann–Whitney–Wilcoxon test and presented as mean and standard deviation or median and interquartile range. All tests were 2-tailed, and *P*-values < 0.05 were considered statistically significant. Statistical analyses were conducted by a biostatistician (Taoufik Ouarrak) using SAS version 9.4 (Statistical Analysis Software, Cary, NC).

## RESULTS

Table 1 summarizes preoperative patient characteristics. The patients in the epicardial group were older (70.3 ± 7.9 years vs 67.3 ± 9.7, *P* < 0.001), with higher body mass index (28.3 ± 5.0 vs 27.1 ± 4.8, *P* < 0.001), lower rate of heart failure (NYHA III/IV 51.8% vs 63.7%, *P* = 0.001), higher incidence of arterial hypertension (79.3% vs 70.3%, *P* = 0.004), higher incidence of diabetes mellitus (27.7% vs 12.6%, *P* < 0.001) and higher EUROSCORE II (5.4 ± 8.4 vs 3.96 ± 6.7, *P* < 0.001).

**Table 1:** Pre-operative patient characteristics

	Epicardial (*N* = 413)	Endocardial (N = 350)	*P*-value
Comorbidities			
Hypertension, %	79.3% (326/411)	70.3% (246/350)	0.004
Age (years)	70.3 ± 7.9, *N* = 412	67.3 ± 9.7, *N* = 350	<0.001
Diabetes mellitus, %	27.7% (114/411)	12.6% (44/350)	0.005
Stroke, %	9.5% (39/411)	6.3% (22/350)	0.10
Sex (female), %	25.4% (105/413)	34.0% (119/350)	0.010
BMI (kg/m^2^)	28.3 ± 5.0, *N* = 411	27.1 ± 4.8, *N* = 350	<0.001
NYHA III/IV, %	51.8% (198/382)	63.7% (207/325)	0.001
AF classification, %			
Paroxysmal	61.5% (254/413)	49.4% (173/350)	<0.001
Persistent	22.8% (94/413)	26.3% (92/350)	0.26
Long-standing persistent	15.7% (65/413)	24.3% (85/350)	0.003
mEHRA, %			
I/IIa	60.1 (170/283)	42.7% (122/286)	<0.001
IIb-IV	39.9% (113/283)	57.3% (164/286)	<0.001
Underlying cardiac d*isease, %*	94.9% (390/411)	98.3% (342/348)	0.012
Coronary artery disease	50.6% (209/413)	13.2% (46/349)	<0.001
Valvular heart disease	45.3% (187/413)	82.2% (287/349)	<0.001
Other	7.5% (31/413)	4.3% (15/349)	0.064
Echocardiographic parameters, %			
LVEF, %	53 ± 12, *N* = 409	54 ± 12, *N* = 348	0.22
LA (mm)	47 ± 9, *N* = 316	49 ± 10, *N* = 295	0.029

Values are expressed as mean ± standard deviation or *n* (%).

AF: atrial fibrillation; BMI: body mass index; LA: left atrium; LVEF: left ventricle ejection fraction; mEHRA: the modified European Heart Rhythm Association symptom classification for atrial fibrillation; NYHA: The New York Heart Association Classification of Heart Failure.

Most of the patients in the epicardial group presented with paroxysmal AF (epicardial 61.5% vs endocardial 49.4%, *P* < 0.001). Also, 60.1% of patients in the epicardial group vs 42.6% of patients in the endocardial group presented with no to mild symptoms (mEHRA I-IIa), related to AF (*P* < 0.001).

Table 2 illustrates periprocedural details. In the epicardial group, coronary artery bypass grafting (CABG) was the most frequent procedure, performed in 57.8% of patients. Aortic valve replacement/repair was performed in 40.8% of patients and mitral valve replacement/repair in 13.6% of patients. LAA exclusion was completed in 385 patients in the epicardial group (93.4%). In the epicardial group, 392 patients (95.6%) underwent radiofrequency ablation. Pulmonary vein isolation performed in 259 patients (69.3%) was the treatment method most practiced in the epicardial group.

**Table 2:** Peri-procedural details

	Epicardial (*N* = 413)	Endocardial (*N* = 350)
Baseline procedure, %		
CABG	57.8% (238/412)	17.4% (61/350)
Aortic valve replacement/repair	40.8% (168/412)	20.0% (70/350)
Mitral valve replacement/repair	13.6% (56/412)	76.3% (267/350)
Tricuspid valve replacement/repair	4.6% (19/412)	19.5% (68/350)
Energy source, %		
Radiofrequency ablation	95.6% (392/410)	2.9% (10/350)
Cryo-energy	5.6% (23/410)	97.7% (342/350)
Linear concept, %		
Box isolation	35.8% (134/374)	93.5% (317/339)
PVI	69.3% (259/374)	39.5% (134/339)
Left atrial lesion set	15.2% (57/374)	75.2% (255/339)
Right atrial lesion set	5.1% (19/374)	23.3% (79/339)
Left atrial appendage closure (%)	93.4% (385/412)	91.4% (320/350)

Values are expressed as *n* (%).

CABG: Coronary Artery Bypass Grafting; PVI: pulmonary vein isolation.

In the endocardial group, mitral valve repair/replacement was the most frequent procedure, performed in 76.3% of patients, followed by aortic valve repair/replacement performed in 20% of patients. LAA exclusion was completed in 320 patients (91.4%) who underwent endocardial surgical ablation. In the endocardial group, cryoablation was performed in 342 patients (97.7%) and completion of a “box-lesion,” performed in 317 patients (93.5%), was the most practiced ablative strategy.

### In-hospital outcomes

Table 3 summarizes postoperative in-hospital outcomes. Upon discharge, 32.3% (*n* = 109) of patients in epicardial and 24.0% (*n* = 72) of patients in the endocardial group showed recurrence of AF. The in-hospital mortality rate was 2.2% (*n* = 9) in the epicardial and 2.9% (*n* = 10) in the endocardial group. Moreover, 8 patients in the epicardial group and 3 patients in the endocardial group suffered a stroke postoperatively. The mean duration of hospital stay was 13 days (range: 10–16) in the epicardial group and 12 days (range: 9–17) in the endocardial group; 20 patients (4.9%) and 28 (8.0%) patients required a new permanent pacemaker in the epicardial and endocardial groups respectively. Table 4 summarizes 1-year follow-up outcomes.

**Table 3:** Postoperative intra-hospital outcomes

	Epicardial (*N* = 413)	Endocardial (*N* = 350)
Rhythm at the discharge, %		
Sinus rhythm	61.0%	71.1%
Atrial fibrillation	31.3%	21.9%
Other	7.8%	7.0%
Complications (intra-hospital), %		
Stroke	2.0%	0.9%
Myocardial infarction	0.0%	1.1%
Renal insufficiency	4.4%	0.6%
Cardioversion,%	14.9% (61/410)	17.2% (60/349)
New devices (intra-hospital), %	10.7% (44/410)	10.6% (37/349)
VVI Pacemaker	1.0%	1.7%
DDD Pacemaker	3.9%	6.3%
Anticoagulation at discharge, %	85.4%	89.1%
Anti-arrhythmic drugs, %		
Class I	2.5%	2.3%
Class II	73.6%	76.8%
Class III	35.3%	19.2%
Mortality, %	2.2%	4.6%

Values are expressed as *n* (%).

**Table 4:** One-year outcomes

	Epicardial (*N* = 413)	Endocardial (*N* = 350)
Follow-up	97.1% (401/413)	96.9% (339/350)
Time until follow-up, days	453.0 (394.0, 579.0)	441.0 (385.0, 549.0)
Complications (post-hospital)		
Stroke	1.1%	2.3%
Pulmonary vein stenosis	0.0%	0.0%
Atrioesophageal fistula	0.0%	0.0%
Anticoagulation (post-hospital)	71.6%	61.1%
AAD (post-hospital)		
Class I	2.7%	1.9%
Class II	85.5%	83.3%
Class III	7.4%	6.4%
Re-Ablation	2.6% (9/352)	2.3% (7/303)
Re-Cardioversion	8.0%	7.9%
One-year procedural success	58.4%	62.2%
Mortality		
Mortality within 365 days after ablation	7.7% (31/401)	5.0% (17/339)

Values are expressed as median (inter quartile range) or *n* (%).

### One-year outcomes: epicardial group

The median follow-up was 15.1 months (interquartile range 13.1– 19.3 months). At follow-up, complete datasets were obtained from 409 patients (97.1%). Details regarding arrhythmia monitoring are provided in Table 5.

**Table 5:** Arrhythmia monitoring at 1-year follow-up—epicardial group

Epicardial group (*N* = 413)	1-year success (*N* = 223)	AF at follow-up (*N* = 159)	*P*-value
Arrhythmia monitoring			
12-lead ECG	85.2% (173/203)	86.9% (133/153)	0.65
24 h ECG	29.1% (59/203)	26.8% (41/153)	0.64
Cardiac implantable device	3.9% (8/203)	5.2% (8/153)	0.56

Values are expressed as *n* (%).

ECG: electrocardiogram.

The overall 1-year procedural success was documented in 58.4% of patients (*n* = 223), and 10.1% of the patients with documented 1-year success in this group continued to use antiarrhythmic drugs of class I or III during follow-up.

In addition, 61.9% (*n* = 146) of preoperative paroxysmal AF patients achieved 1-year procedural success, compared to 52.7% (*n* = 77) in non-paroxysmal AF patients. Notably, following epicardial ablation, 62.2% of patients (vs 25.4% at baseline) reported an improvement in mEHRA I (*P* < 0.001).

The 1-year mortality rate was 7.7% (*n* = 30). A stroke after discharge was reported in 1.1% (*n* = 4) of patients. Also, 28 patients (8.0%) required at least one synchronized electric cardioversion; 9 patients (2.6%) underwent re-do ablation mainly due to atrial flutter/tachycardia. Severe adverse ablation-related complications (pulmonary vein stenosis, atrioesophageal fistula) were not reported in any patient in this group.

### One-year outcomes: endocardial group

The median follow-up was 14.7 months (interquartile range 12.8–18.3 months). Details regarding arrhythmia monitoring are provided in Table 6.

**Table 6:** Arrhythmia monitoring at 1-year follow-up—endocardial group

Endocardial group (*N* = 350)	One-year success (*N* = 201)	AF at follow-up (*N* = 122)	*P*-value
Arrhythmia monitoring			
12-lead ECG	80.3% (147/183)	77.5% (93/120)	0.55
24 h ECG	43.7% (80/183)	39.2.8% (47/120)	0.43
Cardiac implantable device	4.9% (9/183)	5.0% (6/120)	0.97

Values are expressed as *n* (%).

ECG: electrocardiogram.

One-year procedural success was documented in 62.2% of patients (*n* = 201). 5.3% of the patients with documented 1-year success continued to use antiarrhythmic drugs of class I or III during follow-up. Of the patients with preoperative paroxysmal AF, 66.9% had a 1-year procedural success (*n* = 107).

Among the patients with preoperative non-paroxysmal AF, 57.7% (*n* = 94) achieved 1-year procedural success; 65.9% of patients (vs 10.7% at baseline) reported mEHRA I (*P* < 0.001). The 1-year mortality rate was 5.0% (*n* = 17). A stroke after discharge was reported in 2.3% (*n* = 7) of patients. In addition, 24 patients (7.9%) required at least one synchronized electric cardioversion, and 7 (2.3%) epicardial patients underwent re-do ablation mainly due to atrial flutter/tachycardia. Severe adverse ablation-related complications (pulmonary vein stenosis, atrioesophageal fistula) were not reported in any patient in this group.

## DISCUSSION

The CASE-AF Registry is the most extensive nationwide database documenting ablative procedures performed surgically. Each participating centre operates autonomously, employing various ablative techniques, energy sources, medical treatments and post-treatment follow-up protocols. The data management and analysis are independently supervised by The Institute for Heart Attack Research.

Due to these factors, the CASE-AF Registry offers a dependable and comprehensive insight into the surgical practices employed for treating AF across Germany.

The overall 1-year procedural success was 58.4% after epicardial and 62.2% after endocardial surgical ablation consistent with findings in previously published manuscripts [8–11]. In the epicardial group, 61.9% of patients with preoperatively paroxysmal AF and 52.7% with preoperatively non-paroxysmal AF demonstrated 1-year success. In the endocardial group, 66.9% of patients with preoperatively paroxysmal AF and 57.7% with preoperatively non-paroxysmal AF demonstrated 1-year success. It is imperative to underscore the critical importance of achieving a comprehensive lesion set during surgical ablation, as this could directly impact the success of the procedure. The relatively small number of patients who underwent bi-atrial surgical ablation, along with those with incomplete left atrial lesion sets in our registry, may have contributed to suboptimal ablation outcomes.

The limited arrhythmia monitoring in our study, primarily through 12-lead ECG, may have led to undiagnosed asymptomatic AF recurrences, potentially causing an overestimation of the success rate in our research. Therefore, we suggest that future trials contributing to the ablation argument should aim for a more homogeneous, intensive monitoring technique to make the results more reliable. However, it is essential to recognize that the absence of AF-related symptoms does not confirm the absence of AF itself. It was found that asymptomatic AF occurred 12 times more frequently than symptomatic AF when Holter monitoring was used for follow-up [12]. Additionally, the DISCERN-AF study demonstrated that increased monitoring intensity led to higher AF detection rates [13].

A particularly promising finding in our study was the substantial enhancement in clinical status, as evidenced by notable mEHRA improvement in both patient groups. Our analysis unveiled a distinct link between the cumulative number of symptom-free patients in both patient groups and the comprehensive attainment of freedom from AF at the 1-year follow-up. Only 3 studies [14–16] used the RAND 36-item Health Survey assessment tool (SF-36) to assess health-related quality of life after concomitant surgical ablation. von Oppel *et al.* [15] and Van Breugel *et al.* [16] noted a significant increase in physical functioning from preoperative to 1-year postprocedural, but the difference between patients who had concomitant surgical ablation done, and the control group was not statistically significant. Chernyavskiy *et al.* [17] found significant improvement in most RAND 36-item Health Survey assessment tool (SF-36) domains for the patients who had concomitant surgical ablation (CABG + pulmonary vein isolation and CABG + modified Maze) done, compared to patients who underwent CABG only at 1-year follow-up. Berkowitsch *et al.* [14] proposed that this phenomenon might be attributed to the placebo effect, where patients report fewer symptoms due to the belief that their treatment has been successful. Our viewpoint agrees with Chernyavskiy *et al.* [17] suggesting that the improved quality of life after cardiac surgery with simultaneous surgical ablation results not only from curing the underlying disease but also from freedom of AF. Surgeons have expressed concerns about the potential risks of surgical ablation in AF patients, especially in cases of very brief or permanent AF or by AF patients presenting with sinus rhythm before surgery. Previously published manuscripts from the CASE-AF Registry showed that concomitant surgical ablation in AF patients presenting with sinus rhythm before surgery is safe and has a low perioperative risk profile and should be carried out with almost no exception [18]. Rankin *et al.* in 2018 [19] showed that in patients with AF who go for mitral valve surgery, it appears that performing concomitant surgical ablation is associated with a reduced risk-adjusted operative mortality when compared to patients without concomitant surgical ablation. The milestone Cochrane systematic review conducted by Huffman *et al.* in 2016 comprehensively summarized the significant literature on the topic. The review concluded that concomitant AF surgery significantly reduces the risk of recurrent arrhythmias by half with no differences in all-cause mortality or early mortality. However, it is also found that such surgery increases the risk of the need for post-procedural permanent pacemaker implantation and renal failure post-procedural [8].

Following concomitant surgical ablation, the rates of postprocedural permanent pacemaker implantation rate reported in the literature vary widely, with figures ranging from 0.8% to as high as 23.3% [20]. This elevated risk is believed to result from underlying sinus node dysfunction, which becomes apparent when AF-related reentrant circuits are successfully terminated through ablation [21, 22]. Importantly, the indication of permanent pacemaker implantation rate after surgical ablation seems to be more pronounced with performed bi-atrial Cox Maze-IV lesions compared to left atrial or pulmonary vein isolation procedures [9, 19]. This evidence led to the ESC/EACTS downgrading their recommendation for concomitant ablation from class I to class IIA [6]. We have a relatively low rate of postprocedural pacemaker implantation, 4.9% and 8.0% in the epicardial and endocardial group, respectively. This can be partly attributed to the relatively low number of patients who underwent bi-atrial surgical ablation, in our registry. In our view, this should not dissuade clinicians from considering AF ablation if the heart team deems it to be in the patient's best interest. The relatively low rate of perioperative stroke observed in our study, 2.0% in the epicardial group and 0.9% in the endocardial group can be partly attributed to the extensive LAA exclusion procedures. We achieved an impressive LAA isolation rate of approximately 90%. A meta-analysis by Tsai *et al.*, which involved 3653 cardiac surgery patients who underwent surgical ablation, found that the group with additional LAA isolation experienced significantly fewer perioperative strokes, with no increased mortality or higher rates of reoperation due to bleeding [23]. Additionally, Cox et al. demonstrated that resecting or completely closing the LAA significantly reduced the risk of perioperative stroke and nearly eliminated the long-term stroke risk [24].

The in-hospital and 1-year all-cause mortality rates in both groups fall within the ranges previously reported in other studies [9]. Additionally, major adverse cardiac and cerebrovascular events rates in both groups were also consistent with previously reported findings [1, 9]. Our study primarily assessed short-term outcomes. However, Kowalewski *et al.* [25] demonstrated that concomitant surgical ablation led to enhanced long-term survival irrespective of baseline surgical risk.

### Limitations

Two key challenges frequently encountered in observational studies are potential selection bias and unmeasured or uncontrolled confounding variables. For instance, the CASE-AF Registry lacks data on individuals with AF who underwent cardiac surgery but did not receive AF ablation. Furthermore, the registry does not capture the association between rhythm outcome and the results of heart valve surgery, an important factor that could confound treatment efficacy and patient outcome. Serial and Holter ECGs for arrhythmia monitoring post-ablation provide inaccurate rhythm coverage after ablative procedures and appreciably limit the ability to adequately interpret outcomes at 1-year follow-up. Also, our results reflect only the short-term outcomes and do not include long-term follow-up.

## CONCLUSION

Both surgical concomitant ablation approaches, endocardial and epicardial, demonstrate safety and do not elevate the post-procedural complication rate. During the 1-year follow-up, significant improvements in patient clinical status were observed in both groups. Although the procedural success rate was deemed satisfactory in both groups, there is still room for improvement. Expanding ablative line concepts could potentially lead to achieving better outcomes. Prioritizing adequate cardiac rhythm monitoring is essential for ensuring higher quality data acquisition.

## Data Availability

The anonymized data used to support this study’s findings may be released upon application to Ms. Belgin Özdemir, who can be contacted at oezdemir(at)stiftung-ihf.de.

## Notes

Presented at the 37th Annual Meeting of the European Association for Cardio-Thoracic Surgery, Vienna, Austria, 4–7 October, 2023.

## ABBREVIATIONS

AF: Atrial fibrillation
CABG: Coronary artery bypass grafting
CASE-AF: The German CArdioSurgEry Atrial Fibrillation Registry
ECG: Electrocardiogram
EUROSCORE II: The European System for Cardiac Operative Risk Evaluation II
LAA: Left atrial appendage
mEHRA: The modified European Heart Rhythm Association symptom classification for atrial fibrillation
NYHA: The New York Heart Association Classification of Heart Failure
